# 

**Published:** 1895-09-14

**Authors:** 


					Se*t. 14, 1895.
THE HOSPITAL,
A Xtj __
CONTENTS.
405
*h7Re?^a**d Memorial -
n?teioa 'of Maata ?
406
st*e?tIn i,ratlon of Plants -
?o.ea anrt w??ns for Charities
??-es ? ions
410
Medical pilew*  *10
PrenS^8^6?? and. Hospital Clinlos?
PrewToi^S' Bna ?OBPltal Cllnioa-
Po*t< ? MHlformation in the Newly Born-
-Case of Mento-
p0,f": m sae newiy jjorn?uase or jwento-
Head ?r P8,0^011:?Successful Raising and Rotation of
411
Jrogr
3RFSo .
P*OGF--8 IN-8uRG?RT-
-Appendicitis ?
411
Pfcflno IN OWR0EBT.?.
41S
? Ilf utkjbcoloot
ApM.U?CE8 and Things Medical
416
try i a.
I The Study of Van;-
-IX. A New Rmp in Egypt ?
407
Social Problems.
-Child Lif* in France
408
Annotations. ? A Hospital for Coatbridge?The Powers of the
Shin's Surgeon?A School of Economioi . . .
409
The Institutional Workshop?
Hospital Admi?is*iiitiox.-
-Toronto General Hospital
- *17
The Stout or the Insane from Ycab to Yiaii
418
Practical Department
418
Aboc'd thk Hoar tam
? 419
OOKSTBUCTIOS NOTES
420
Soraps and Gleanings
420
EXTRA SUPPLEMENT.-THE NTJBSIN <J MIRROR,
iae rrurslngf World?London Hospital Nnrs-
'nEf Home?Gnardiarg and District Nursing?A Deserving
?ciety?Rural Dig htheria?Night Nursing in Irish Work.
__^8ea~The Truro District Nurse?TheDrogheda Workhouse
r:Bally?astlo Workhouse?Long Hours of Work?Aberdeen
Nurfing Assoo ation
j Schools for Trained STursea: Their Origin
- no Otihh,ui.h?.? V.?The Rioetre and Pitid Sohools *
Elementary Physiology for Nurses. By 0. F. Mae-
shall. M.D., B.Sc., F.R.0.8. TI.?The Oironlstory System
elzi
clxii
? elxiii
- olzir
? olxiv
_  - olx-vi
Dotes and* Queries ? ? ? ? olxvi
(concluded)
Appointments?Minor Appointments
Pt. Olave a Union Infirmary
?'The Ockley System" ? ?
Tbe Book World for Women and ITnrses
The Xrospital" Convalescent Fund

				

